# Long-Term Conservation Management of the Endangered Canarian Lizards *Gallotia simonyi* and *G. bravoana* (Fam. Lacertidae) (2006–2024)

**DOI:** 10.3390/ani16121869

**Published:** 2026-06-17

**Authors:** Miguel A. Rodríguez-Domínguez, Sonia Plasencia-Rodríguez, María M. Suárez-Rancel, Ignacio Domínguez-Espinosa, Albert Martínez-Silvestre, Martha L. Bohórquez-Alonso, Miguel Molina-Borja

**Affiliations:** 1 Centro para la Reproducción e Investigación del Lagarto Gigante de El Hierro, Cabildo de El Hierro, 38911 Frontera, Spain; mrodriguez@elhierro.es; 2 Centro de Recuperación del Lagarto Gigante de La Gomera, Valle Gran Rey, Cabildo de La Gomera, 38870 Valle Gran Rey, Spain; splasencia@lagomera.es; 3 Facultad de Ciencias, Sección Matemáticas, Universidad de La Laguna, 38200 La Laguna, Spain; msuarez@ull.edu.es (M.M.S.-R.); alu0101316420@ull.edu.es (I.D.-E.); 4 Centro de Recuperación de Anfibios y Reptiles de Cataluña, Masquefa, 08783 Barcelona, Spain; crarc-masquefa@outlook.com; 5 Grupo de Investigación “Etología y Ecología del Comportamiento”, Department Biología Animal, Faculty Ciencias, Sección Biología, University La Laguna, 38200 La Laguna, Spain; mlbohor@ull.es

**Keywords:** lizards, conservation, *Gallotia simonyi*, *G. bravoana*, breeding, reintroduction

## Abstract

**Simple Summary:**

Accumulated information is presented on captive breeding in outdoor terraria and on reintroduction efforts for the endangered lizards from El Hierro (*Gallotia simonyi*) and La Gomera (*G. bravoana*) over the past several decades. For individuals used annually as breeding stock, we report species-specific reproductive parameters: the number of eggs laid (NEL) by females, the incubation procedures, and the number of hatched offspring (HO). We compare these metrics between species and, within each species, across years. Statistically significant differences were detected both between species and among years for these variables. Veterinary examinations indicated that individuals were generally in good condition, with only a few showing abnormal traits. The original wild populations of both species still persist, and preliminary estimates of their potential population sizes have been made. Recent reintroduction attempts into natural sites have been conducted, although success has so far been only partial. A stable population of *G. simonyi* persists on a small islet in the north-west of El Hierro, and additional individuals (estimated number pending confirmation) remain at two other reintroduction sites. Early reintroduction attempts for *G. bravoana* were unsuccessful, but some individuals are still present at a more recent release site.

**Abstract:**

*Gallotia simonyi* and *G. bravoana* are large lacertids inhabiting the islands of El Hierro and La Gomera, respectively, in the Canary Archipelago. Both species are critically endangered, but over the last several decades, they have been bred in outdoor terraria (*G. simonyi* since the 1990s and *G. bravoana* since 2000). In this study: (1) we describe all procedures carried out in the breeding centres and quantitatively analyse the long-term trajectory of breeding success throughout the study period; (2) we examine whether any parental individuals or specific pairs had a stronger influence on the number of successfully hatched offspring; (3) we report the trials of reintroducing individuals into the wild on each island in different years; (4) we provide information on several predator (cat-control) campaigns conducted on each island; (5) we detail the veterinary protocols and the results obtained when assessing the health status of breeding lizards; and (6) we report several educational activities carried out on each island. Gravid females laid eggs in suitable laying boxes; the eggs were then kept inside incubators with controlled temperature and humidity until hatching. Breeding produced 1267 offspring during the years considered for *G. simonyi* and 499 for *G. bravoana*. The mean NEL was 8.8 for *G. simonyi* and 5.2 for *G. bravoana*, and the mean HO was 6.4 and 3.54, respectively. Both NEL and HO were significantly higher in *G. simonyi* than in *G. bravoana*. NEL was significantly influenced by species and year, and by female snout–vent length (SVL) as a covariate, but not by male SVL. HO was significantly affected by year and by both male and female SVL, but not by species. There were significantly higher or lower values of both variables in specific years, but no clear long-term trend. Some breeding pairs had a greater influence on the dependent variables. Reintroduction into the wild has resulted in a currently stable population of *G. simonyi* on a small islet off the north-western coast of El Hierro, and some individuals are still present at an inland reintroduction site. For *G. bravoana*, some live specimens have recently been detected at a new reintroduction site. We conclude that: (1) captive breeding has been successfully carried out over the years in both centres; (2) there have been significant differences between the two species in NEL and HO; (3) female SVL was significantly related to both NEL and HO; and (4) reintroduction attempts have been only partially successful in each species. Veterinary monitoring revealed high dehydration tolerance, seasonal fluctuations in microbial flora, previous mineral imbalances that were corrected by improved nutrition, and effective parasite control that maintained overall lizard health. Except for a few individuals, most lizards were in good health.

## 1. Introduction

The lizards of the genus *Gallotia* (Fam. Lacertidae) are endemic to the Canary Islands and are a key taxon in the lizard phylogenetic tree [1]. Within this genus there are four species of large lizards: (1) *Gallotia stehlini* (from Gran Canaria Island), which is not endangered (although the recent introduction of the invasive snake *Lampropeltis californiae* is threatening some local populations) [2]; (2) *G. simonyi* (from El Hierro Island), listed as “Vulnerable” by IUCN [3], although the information provided is not updated; (3) *G. bravoana* from La Gomera Island, listed as “Endangered” by IUCN [4], also not updated; and (4) *G. intermedia* (from NW and SW Tenerife), considered as “Endangered” by IUCN [5]. The three last species, initially considered extinct, were later rediscovered: *G. simonyi* in 1975 [6,7]; *G. intermedia* in 1996 [8], and *G. bravoana* in 1999 [4,9]. These three species are genetically very closely related [10].

*Gallotia simonyi* was widely distributed in the past over El Hierro (Canary Islands [11]). Its original natural habitat is the area known as Risco de Tibataje, a Special Natural Reserve [12]. In 1986, a captive breeding programme was initiated, and some specimens were included in two successive LIFE European projects, which provided funds to continue a recovery plan in subsequent years [13]. The first official recovery plan was established by the Autonomous Government of the Canary Islands in 2004 [14]. Very recently, the Canary Islands Government published a new recovery plan [15].

Six individuals were discovered and captured from a very small wild population of *G. bravoana* in 1999 at Risco de la Merica (a high inland cliff located in southwestern La Gomera [9]). They were initially kept in temporary terraria, and captive breeding was initiated in 2000 [16,17]. The first official recovery plan was published by the Canary Islands government in 2006 [18], and several conservation actions funded by the island’s Cabildo have been undertaken since then (detailed in several sections of the present work).

The first group of *G. intermedia* individuals was discovered in high coastal cliffs in NW Tenerife [8], and four years later, a new population was discovered on Guaza Mountain (SW part of the island) [19]. Several reports on this species have been submitted to the Autonomous Government of the Canary Islands [20], and an undergraduate thesis was completed on the Guaza population [21], but an official recovery plan (not including captive breeding) was not published until 2017 [22].

Over many years (see below the Methods section), captive breeding in outdoor terraria, veterinary monitoring, and several reintroduction attempts have been carried out for both *G. simonyi* and *G. bravoana*. However, according to the most recent IUCN assessment (see above), *G. simonyi* is still classified as “Vulnerable”. Recent data (compiled in the Results section) show that, apart from the original population at Risco de Tibataje, only a small population persists on the islet of Roque de Salmor, and an unknown number of individuals occur in an inland reintroduced population. In the case of *G. bravoana*, it is listed as “Endangered” by the IUCN, and our recently compiled data (see Results) indicate a worrying situation: the original population at Risco de La Mérica shows a reduced number of individuals compared with assessments from previous years, and an unknown number of individuals are still surviving at a recent reintroduction site. By comparing breeding success, veterinary data, and reintroduction outcomes for both species, we aimed to identify potential factors underlying their differing conservation status.

The first aim of this work is, for each species, to determine whether there has been any trend in the number of eggs and hatchlings obtained during the study period. The second aim is to identify whether any specific females, males, or pairs have had a disproportionately large influence on the number of successfully hatched offspring. Based on previous published studies on several lizard species [23,24,25,26,27], we predicted that the number of laid eggs should be related to female (or male) body size. Given that adult snout–vent length (SVL) is smaller in *G. bravoana* than in *G. simonyi*, we also predicted that the number of eggs laid by females would be lower in the former species. A further prediction was that no clear temporal trend in the number of eggs laid or hatchlings produced would be observed. This prediction was based on the heterogeneous characteristics (body size, ages, etc.) of adult lizards participating in reproduction throughout the study period. Considering that most of the lizards used as breeders during these years were born in captivity, we also expected that some sign of inbreeding might appear, at least in some individuals. Regarding reintroduction trials, we expected that released lizards would contribute to the establishment of viable wild populations. We show here that: (1) captive breeding has been successful, although with interannual variation; (2) there were significant differences in NEL and HO between the two species; (3) female SVL had a significant effect on NEL and HO; (4) reintroduction attempts have so far been only partially successful in each species; and (5) except for a few *G. bravoana* individuals that showed some abnormal traits, the lizards generally exhibited good health status.

## 2. Materials and Methods

Reproduction of both species in breeding centres began several decades ago (after 1986 for *G. simonyi* and from 2000 onwards for *G. bravoana*). However, we only had limited access to data for *G. simonyi* during some of the years in the 2006–2024 time period. A similar situation occurred for *G. bravoana* during 2009–2024 (see details in Table 1). The reasons for excluding data were as follows: no reproductive pairs were established by the breeding centre staff; some individuals were given rest periods; and data were missing from some individuals. The data included in the current article were submitted annually to Consejerías de Medio Ambiente (Environmental Departments within each Island Council) in the form of technical reports prepared by several author teams contributing to the present work [references included below in each text section]. Both Cabildos authorised the work performed at each breeding centre and in natural locations of each island.

The present contribution reports on: (1) body size of males and females participating in reproduction at both El Hierro and La Gomera breeding centres over several decades (as mentioned above); (2) the number of eggs laid by females and the number of hatchlings; (3) the relationships between male or female body size and these two variables; and (4) whether there was a relationship between male-to-female SVL index and the number of eggs or hatchlings. We also include: (5) data from several trials in which adult males and females of each species were reintroduced into wild locations on their respective islands; (6) procedures and results of veterinary examinations performed on individuals of each species to assess lizard health status; and (7) specific educational activities carried out on each island to promote science and to communicate the history of each species and the conservation actions undertaken.

### 2.1. Individual’s Maintenance

El Hierro giant lizards (*G. simonyi)* are large lacertids with a maximum SVL of around 200 mm for wild-living lizards [28]—up to 247 mm for males and 218 mm for females kept in terraria (present work). For several decades, groups of males and females have been maintained in separate outdoor terraria (4 × 3 m). These contain leaves and flowers of naturally growing local plants (verode, *Kleinia neriifolia*; calcosa, *Rumex lunaria;* tedera, *Bituminaria bituminosa*), and provide several hiding places in the form of thick cork bark and palm leaves; all terraria are covered with a wire mesh on top to prevent avian predation. Food for the lizards is provided three times per week and consists of local plants (verode, *K. neriifolia*; tedera, *B. bituminosa*; cerrajón, *Sonchus* spp.), supplemented occasionally with alfalfa (*Medicago sativa*) and insects (crickets *Gryllus bimmaculatus* and *Tenebrio* larvae) (see housing details in [29]).

In La Gomera, groups of males and females of *G. bravoana* (maximum SVL = 202 mm for a male and 190 mm for a female; present work) were also kept in separate terraria (6 × 4 m) with growing local plants (similar to those used in El Hierro, and also including balo, *Plocama pendula*). In this case, hiding places consisted of plastic tubes (60 × 16 cm, length × diameter) inserted into ground hollows constructed for this purpose; palm leaves were also placed to provide shade. Food was provided following the same time schedule as for *G. simonyi*.

### 2.2. Veterinary Surveillance

The health control and veterinary monitoring programme was initiated for both species in 2000 and resulted in the publication of scientific studies during the first ten years of the programme. These studies initially addressed veterinary assistance in conservation actions for the La Gomera giant lizard. In the years following the descriptions of physiological parameters, health assessments were repeated approximately every two years with the aim of ruling out diseases that could affect recovery actions. Emerging pathogen screening was introduced in 2022 and has been conducted on randomly selected lizards of both species. Reovirus, adenovirus, and ranavirus were analysed, and the results were consistently negative.

Blood, cloacal, glandular, and tissue samples from different populations of captive lizards were examined using cytochemistry, microbiological culture, molecular assays, and gas chromatography–mass spectrometry. Biopsies and necropsies were conducted, and selected tissues were histologically examined using Hematoxylin–Eosin staining. In general, lizards were found to be in good condition throughout the years analysed [30,31,32,33]. However, congenital malformations (tail vertebral deviations) have been detected in some individuals since 2016 [34]. Moreover, in the last year, we found several individuals of *G. bravoana* showing symptoms of inbreeding, such as skin pigment alterations (ivory colouration) and brachygnathia [35].

### 2.3. Selection of Adults for Reproduction

Adult male and female individuals ranging from four to eleven years old were selected at the beginning of each breeding period to participate in reproduction from among the individuals available at each breeding centre. During the rest of the year, males and females were kept in separate terraria. Each lizard had a microchip (BIO GLASS 8625, Felixcan Syringe Labtag -Mini-, Technology FDX-B, ISO 117845/5 [36], Felixcan, Albacete, Spain) inserted under its neck skin for permanent identification. The corresponding code and SVL, measured each year for each individual, were recorded in an Excel spreadsheet.

Mating pairs were selected according to three requirements: (1) genetic information on the relatedness among individuals was generally not available (except for some individuals of each species in some of the last five years). We therefore used the criterion that males from each pair had a larger body size than that of females; this decision was based on previous publications reporting that, when females select males for mating based on visual traits, the most commonly detected trait is body size [37,38,39]; (2) members of each pair were not directly related (brothers or cousins); and (3) exactly the same mating pairs had not been used in previous years. However, given the limited number of available adult males and females in each breeding centre, some of them had to be used (with different partners) in different years. The same protocol for selecting and maintaining mating pairs was established for both species. Around the first or second week of May each year, each established pair was transferred to separate outdoor terraria (3 × 2 m) to allow them to interact and mate. Details of courtship and copulation patterns and the general reproductive cycle have been published previously for *G. simonyi* [29] and reported for *G. bravoana* [16].

### 2.4. Laying Females, Eggs, and Care of Newborns

Approximately one month after mating, females were manually palpated (by MARD, for *G. simonyi*, and by SP for *G. bravoana*) to confirm the presence of developing eggs. When eggs were detected, each female was transferred to an individual outdoor terrarium without **a** male. Females remained in these terraria until the end of June, when each one was moved to an individual indoor laying terrarium (made from wood, 45 × 50 × 50 cm). Light was provided with a photoperiod of 7L/17D (light beginning at 10:00 h and ending at 17:00 h) using fluorescent tubes with a daylight spectrum, including part of the ultraviolet range (UVA, Reptistar F18W 6500 K, Sylvania, Budapest, Hungary). This photoperiod was set because, in both breeding centres, sunlight reaches the outdoor terraria late in the morning (around 10:00 h during summer). Each terrarium contained a small wooden box (20 × 25 × 35 cm) filled with silica sand. A 40 W bulb was provided as a heat source, and the temperature inside nest boxes ranged between 26 °C and 28 °C with 80% humidity.

Laying boxes were checked daily to determine whether females had laid eggs. When eggs were found, they were carefully transferred to plastic containers (32 × 15 × 7 cm) with a hermetic seal and filled with vermiculite. Eggs were buried (substrate moisture 80–90%) in the same orientation in which they were collected, and the boxes were placed in an incubator at a constant temperature (28–29 °C) and humidity (75–85%). These same temperatures and humidity were used every year [29] and for both species.

After hatching, newborn lizards from each clutch were transferred to wooden terraria (45 × 50 × 50 cm, *G. simonyi*) or coated aluminium terraria (80 × 40 × 40 cm, *G. bravoana*). There, they were fed leaves, flowers, buds, and seeds of *B. bituminosa*, *K. neriifolia*, *Rumex lunaria*, pumpkin flowers, lettuce, *Tenebrio molitor* larvae, and small crickets (*Gryllus bimaculatus*), with water provided ad libitum. After a 40-day period in these terraria, during which the young were checked to control for any sign of disease or injury, they were moved to outdoor terraria (328 × 457 × 172 mm). The same procedures were followed to monitor female pregnancy and to manage laying, egg care, and newborn care in *G. bravoana*.

### 2.5. Reintroductions

In recent decades, several trials have been carried out to reintroduce captive-bred individuals (three–four years old) of each species into a few natural localities on the corresponding islands. The reintroduction at specific sites within each island over several years was decided by the Environmental Office staff of each Cabildo, with the assistance of a professional climbing team. The suitability of each site was determined using the following criteria: the site was very difficult for humans to access and, when possible, was free of feral cats [40]. Reintroduction trial data have been available since 1999 for *G. simonyi* and since 2013 for *G. bravoana*. Compiled data on reintroduction dates, sites, and the number of adult individuals released were gathered from our own files and from those of authors who carried out the reintroductions in the field or performed subsequent evaluation in these areas.

### 2.6. Data Analysis

The number of eggs laid (NEL) and successfully hatched offspring (HO) were recorded for each year and parental pair. We also measured the SVL of all parents and calculated the SVL difference within each pair.

Generalised Linear Mixed Models (GLMMs) were initially applied with parental code as a random effect to account for individual influence on NEL and HO. However, as individual variance was negligible (see Results), we proceeded with the Generalised Linear Models (GLMs) [41,42].

Separate GLMs were fitted for each dependent variable using a Poisson distribution and log link function [43], with species and year as fixed factors and male and female SVL as covariates. An additional GLM was fitted using the male-to-female SVL difference as a covariate. Model selection was based on the Akaike Information Criterion [41]. The sample size was larger for *G. simonyi* than for *G. bravoana*. However, for the comparison between the two species in the set of GLMs fitted in this study, the distribution of observations corresponded to an approximate 60/40 split. This level of imbalance falls well within the range generally considered acceptable for GLM-based inference. Limitations associated with sample size imbalance typically arise under substantially more extreme conditions (e.g., strongly skewed distributions such as 90/10 or higher), which may lead to reduced precision or undue influence of the larger group. Given the relatively balanced nature of the dataset, all models were fitted jointly, without the need for weighting or stratification based solely on species sample size.

Finally, Cook’s distances [44] were calculated to identify potentially influential parental pairs in the analysis.

## 3. Results and Discussion

### 3.1. Morphological Traits of Lizards

**Figure 1:**
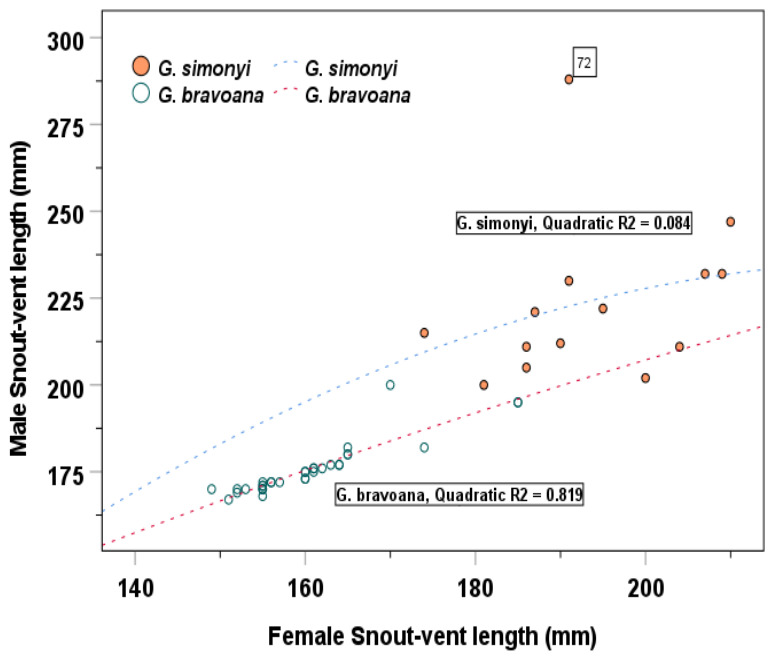
Relationship between female (*x* axis) and male SVL (*y* axis) within each species in one of the sampled years. Dotted lines: fitted regression lines. Point marked with 72 corresponds to a particular pair of *G. simonyi* in which the male SVL was much larger than that of the female.

In Table S1a, b, we present the basic SVL statistics from the 198 males and females of *G. simonyi* and the 133 individuals of *G. bravoana* that participated in reproduction in each of the years for which data were available. In *Gallotia* species, sexual maturation can be attained between three (females) and four (males) years [45]. In every sampled year, males always had a longer SVL than females (Table 1), and in most years, there was a significant linear relationship between female and male SVL (Figure 1). A relationship has been found between lizard body size and reproductive success in some species [23]. Therefore, using adult lizards with the longest SVL should, in theory, contribute to greater reproductive output. However, several factors may influence reproductive success in lizards, such as food availability [46,47], environmental temperatures, and others (see below).

As specified in Methods, we selected the breeding pairs of each year such that males had a longer SVL than females. This decision was based on the literature showing that female lizards rarely seem to select males for mating based on a specific trait [37,48,49]; only in some cases are male coloration or body size actively selected by females of some Squamata [38,49,50]. See below (end of Section 3.2) for the influence of female SVL on the number of eggs laid and hatched offspring.

### 3.2. Numbers of Laid Eggs and Hatched Offspring

In Table S2a,b, we present basic statistics for the number of eggs laid (NEL) and hatched offspring (HO) obtained from the different mating pairs of each species in each of the sampled years. Considering data from all years in each species, the mean NEL was 8.8 for *G. simonyi* and 5.2 for *G. bravoana*, and the mean HO was 6.4 and 3.54, respectively. Both NEL and HO were significantly higher in *G. simonyi* than in *G. bravoana* (Kruskal–Wallis test; H = 93.41 and 46.40, respectively, for each variable; *p* < 0.0001 in both cases). Within each species, there was a significant correlation between NEL and HO (Spearman’s rho = 0.674 and 0.686, respectively, for *G. simonyi* and *G. bravoana*; *p* < 0.001 in both cases).

The GLMM used to detect the possible effect of individual code as a random factor on each dependent variable showed that it had a negligible influence: both dependent variables had zero variance except for HO in *G. simonyi*, for which the effect was non-significant (male codes: z = 0.544, *p* = 0.586; female codes: Z = 1.345, *p* = 0.178).

GLM analysis showed that NEL was significantly influenced by species and year, and by female SVL as a covariate, but not by male SVL (Table 1). When we removed male SVL as a covariate, the AIC value was lower (Table 1), indicating that the corresponding model was more appropriate. These results are consistent with the previously reported effect of female SVL on the number of eggs laid in lizards; NEL has commonly been found to be positively and significantly related to female SVL or body mass (BM) in other lacertid species [51], including a previous analysis in *G. simonyi* [29] and a phylogenetically based study on the genus *Gallotia* [24].

There was also a significant effect of year, male and female SVL on HO, but not species (Table 2). When the factor species was not considered, the AIC value was lower, indicating, again, a better model (Table 2).

No conclusions could be drawn on any dependent variable when considering male-to-female SVL difference as the only covariate (Table 3).

The effect of the random variable (individual code) could not be analysed as many individuals only appeared once in the data. When considering reproductive pairs, some of them showed a greater influence on the dependent variables (greater Cook distances; Table 4).

**Figure 2:**
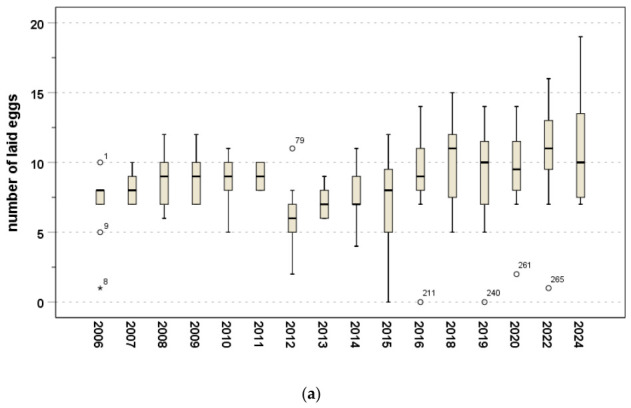

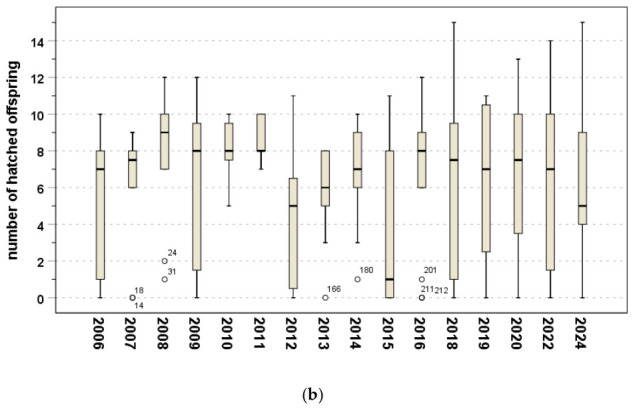
Medians, 75% percentiles, and minimum and maximum values of the number of eggs laid (**a**) and hatched offspring (**b**) in *G. simonyi* from each sampled year. Case numbers and points outside bars are extreme values, and * is an outlier.

**Figure 3:**
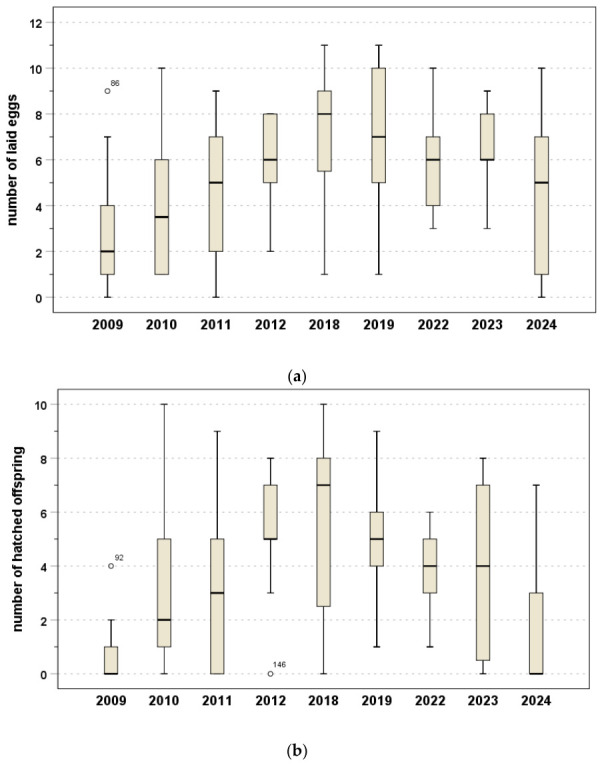
Medians, 75% percentiles, and minimum and maximum values of the number of eggs laid (**a**) and hatched offspring (**b**) in *G. bravoana* from each sampled year. Case numbers and points outside bars are extreme values.

There was inter-year variation in the number of laid eggs and hatched offspring in each species but no clear trend (Figure 2a,b and Figure 3a,b). Values of NEL were higher in 2019, 2023, and 2024 for *G. simonyi* and during 2012–2018 for *G. bravoana* (Figure 2a and Figure 3a). Values of HO were higher in 2010 and 2011 for *G. simonyi* and in 2018 for *G. bravoana* (Figure 2b and Figure 3b).

The separate GLM analysis of NEL and HO within each species, considering only the factor year, showed significantly lower NEL in 2006, 2012, 2013, and 2015 (Table 5) and lower HO in 2012 and 2015 for *G. simonyi* (Table 5). For *G. bravoana*, NEL was significantly lower in 2009 and significantly higher in 2018 and 2019, while HO was significantly lower in 2009 and significantly higher in 2012, 2018, and 2019 (Table 6).

As shown above, in most cases, the number of hatched offspring was lower than the number of eggs laid. The proximal causes of failure of some offspring to hatch were: reduced egg size, minimal increase in egg weight, or absence of any embryo [29]. Moreover, inter-annual variation in HO may be due to several factors such as differences in the specific pairs established for mating, which implies potential effects of (unknown) behavioural mate incompatibility, underlying female gamete selection, or genetic distance between paired individuals [37,52]. Inter-annual variation in NEL and HO may, of course, also depend on climate variation (e.g., different timing of adequate environmental temperatures to initiate spring activity [26,49,53,54].

The fact that the various factors involved in the reproductive output of established pairs throughout the study period resulted in reduced values for *G. bravoana* in 2024 was a source of concern. This was known by the official staff of the breeding centre and was also reported to the Environmental Office of the Cabildo of La Gomera (officially responsible for the species’s recovery plan). Several improvement measures were implemented (including providing additional nutritional supplements and considering greater genetic distance between parental pairs), and as a result, NEL and HO increased in 2025 (data not fully analysed yet).

In some other lizard species, hatching success and survivorship of free-living juveniles have also been associated with the number of males a female mate with [55,56]. Multiple paternity has been detected in many lizard species [57]. Due to the limited number of available adult males, we could not assess the effect on female fecundity of providing more than one male partner per reproductive season. Moreover, due to constraints in the breeding centres, we could not determine whether changing male partners each year affected female fertility. Under field conditions, female lizards of other species can have more than one male partner per breeding season, and this has an impact on their reproductive success [58,59].

Male body size has been found to be a good predictor of mating success in field studies of many lizard species [48,49]. However, it is not clear whether this is mediated by active female choice or is a consequence of successful males overlapping many female territories, or extra-territorial matings by males.

The possible influence of any other specific male trait remains to be tested. In the ornate dragon *Ctenophorus ornatus*, relative male head size—an important secondary sexual trait in lizards—influenced hatching success [48], but in this case, male reproductive success was estimated under field conditions and with males having more than a single female partner. Some mammal and bird females invest more in eggs or offspring of one sex, or change the sex ratio of their offspring, when paired with males bearing attractive traits [60,61]; however, the underlying mechanisms are not yet known.

Other factors may also play a role in male and female reproductive success. For example, in *Lacerta agilis*, males with higher genetic similarity to the female partner were shown to sire a lower proportion of her offspring than more distantly related males [52], suggesting that there may be female selection of the sperm that fertilises her eggs. As female physiology is responsible for allocating reserves to eggs [62], females must respond—in ways that are still unknown—to the traits of their male partners, resulting in higher egg investment and, therefore, a higher number of successfully hatched offspring when paired with attractive males. Selection of fertilising sperm at the level of the ova may be an alternative or complementary mechanism for increasing embryo viability and, therefore, hatching success [52]. In our case, some genetic analyses have been carried out in recent years [63,64,65]; however, data analyses are still underway with the aim of elucidating the potential influence of male–female genetic dissimilarity on NEL, HO or juvenile survival.

### 3.3. Original Habitats and Reintroductions of Lizards into Other Natural Habitats

#### 3.3.1. Estimation of Lizard Numbers in the Original Habitat (*G. simonyi*)

Population size in the original habitat of Risco de Tibataje was estimated using the Schnabel method in 2007 and 2019, yielding 354 and 834 lizards, respectively [66,67]. Estimates of lizard numbers prior to 2007 were obtained by different authors, who reported figures ranging between 100 and 200, and 900 lizards in this habitat (several references cited in [68]). The highest estimates obtained are broadly similar. However, given the different methodologies and total areas surveyed in the various studies, a strict comparison cannot be made, and, therefore, we cannot determine population size with precision or describe its dynamics over the study period.

##### Reintroduction Trials Outside the Original Habitat

In Table S3a, we summarise the number of captive-bred individuals reintroduced at several natural localities on the island since 1999, together with data from subsequent evaluations at each site.

**Figure 4:**
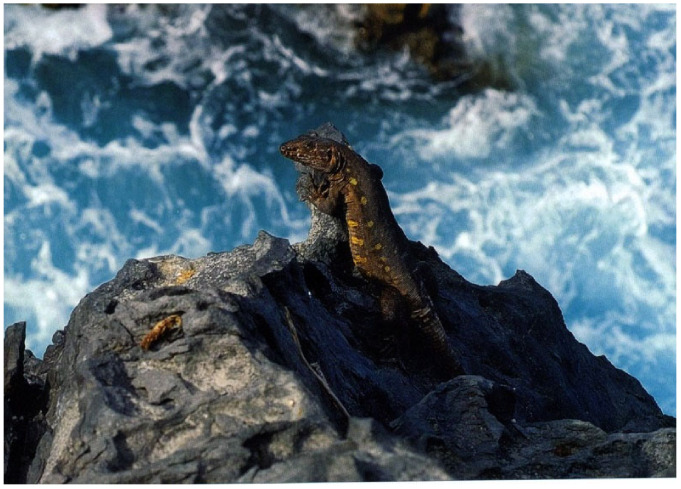
An adult female of *G. simonyi* from Roque Chico Salmor. Photograph by M.M.B.

In 1999 and 2000, a total of 36 lizards were introduced onto a small islet (Roque Chico de Salmor) located off the north-western coast of El Hierro (Table S3a [69,70]). A visit to this population in 2004 confirmed the presence of live lizards, including some juveniles [71,72]. Later, an assessment of lizard abundance at this site estimated a population of 126 individuals (95% confidence limits: 62–254) in 0,1 ha (Figure 4; [73]). Other groups of lizards were reintroduced at two additional sites on the island (La Dehesa and El Julan in 2001 and 2003 [73], respectively), but a field survey in 2004 detected only two individuals at La Dehesa and none at El Julan ([71], Table S3a). In 2008, an evaluation using the Schnabel method again estimated only very few lizards at both sites [73]. It was therefore concluded that these two populations were not able to persist on their own.

Since 2013, three sites, different from the previously surveyed, were selected for new reintroductions (Table S3a) [40]. In two of them (Punta Arelmo and Corrales de Agache), 35 and 33 lizards, respectively, were released in 2013 by staff from the Cabildo of El Hierro (Table S3a). In 2019, the climbing team was able to access the second area and, although they could not estimate population size at the time, they collected up to 75 recent lizard faecal samples [74]. This indicated that individuals from the 2013 release were still alive in the area and, therefore, it was decided to reinforce that population by releasing 50 additional lizards in 2020 [75]. A new visit to this site in 2023 again confirmed the presence of fresh lizard faeces, and the staff from the Environmental Council of the Cabildo of El Hierro decided to release 71 more lizards in November 2024 [76]. A census (which could not be conducted earlier due to logistical and funding constraints) is scheduled for the next few months of 2026 in this area to obtain an estimate of the actual population size. In a third location (Punta Miguel), 46 lizards were released in 2016 [77], but in 2018, no signs of lizard presence were detected [78].

Some groups of lizards were trained prior to reintroduction at Corrales de Agache to recognise models of a kestrel and a cat [79]. An evaluation of those lizards could not be conducted in situ due to logistical and funding constraints; however, at least some individuals were confirmed to still be living there (Table S3a). Of course, training for predator recognition can only be considered one of the factors that may have affected the survival of those lizards; the absence of cat predation or the adequacy of resources at the release site may have played a role [80,81].

#### 3.3.2. Estimation of Lizard Numbers in the Original Habitat (*G. bavoana*)

A field survey in 1999 estimated a population of around 160 lizards in isolated patches totalling less than 20 km^2^ [82]. In 2016, a visit by a climbing team to the inland cliff of Risco de la Mérica detected a reduction in fresh lizard faeces (compared with those found on previous dates) in the areas where living lizards were previously present [83].

##### Reintroduction Trials Outside the Original Habitat

**Figure 5:**
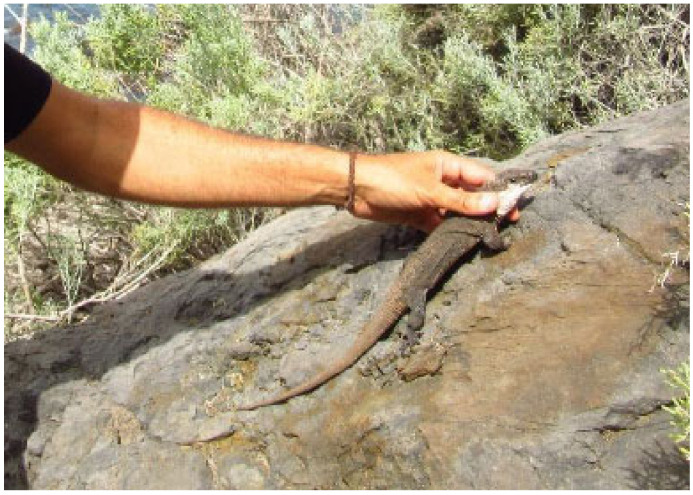
An individual of *G. bravoana* being released into a wild area of La Gomera during 2022. Photograph by Tenerife-Vertical.

In Table S3b, we compile data on the dates and number of lizards reintroduced into the natural environment at several localities on La Gomera Island for this species since 2013 (Figure 5)**,** together with some data from subsequent evaluations at each site. Before that date, an experimental release was conducted with six adult lizards in Los Órganos (Table S3b, [17]).

The first reintroductions of individuals of this species into the wild took place in 2013 and 2014, in Oroja (SE, 18 lizards) and Los Organos (NE, 135 lizards, Table S3b), respectively. However, they were not successful, as in the second site, an evaluation performed in 2016 did not detect any lizards or signs of their activity [83]. A recent reintroduction of 83 lizards was carried out at a different site, Corrales de Heredia (SW of the island) [84]. A census evaluation is scheduled to be undertaken in the coming months of 2026.

#### 3.3.3. Factors Affecting Reintroduction Success

Current results for both species show that reintroduction trials have only been partially successful in establishing self-sustaining lizard populations at some of the selected reintroduction sites. For example, in the case of *G. simonyi*, a stable population has been achieved from lizards reintroduced onto the small islet (Roque Chico de Salmor) situated close to the north-west coast of El Hierro. There, the most recent evaluation of lizard abundance estimated around 127 individuals [73]. More recently, an unknown number of lizards were still alive in Corrales de Agache (Martín-Carbajal and Martínez-Iglesias, pers. comm.). In the case of *G. bravoana*, the situation is more critical, as, apart from the individuals kept at the breeding centre (around 300 individuals), there are only two wild populations: the original population in Risco la Mérica and a new reintroduction site (the number of lizards remains unknown).

Some preliminary population viability analyses (PVA, using Vortex 1.0 software) showed for each species that a higher probability of long-term survival of released lizards would occur only when the initial reintroduced population comprised one hundred individuals (or more) and when no strong negative environmental effects impacted that population (our own unpublished data).

In the lizard *Ameiva polops*, 57 adults were translocated to a Caribbean island after their main predator, the mongoose (*Herpestes auropunctatus*), was eradicated. The lizards were initially translocated to 100 m^2^ open-top enclosures on parts of the island, which were opened after 71 days; five years later, the population had increased to 1473 individuals [80]. This procedure is known as a soft-release, and we have recommended its use for both *Gallotia* species in technical reports previously submitted presented to each Island Cabildo. In *Psammodromus algirus* (phylogenetically close to *Gallotia* [1]), the outcome of introducing two captive-bred cohorts (178 and 187 lizards in two consecutive years) was monitored in two woodland fragments of Spain [81]. After 4 years, a stable population was recorded in one of the areas that previously lacked a viable population of this species. Results from both of these studies show that lizard reintroductions can be successful when limiting factors are properly addressed. The selected wild habitats for releasing *G. simonyi* and *G. bravoana* apparently provide key resources for lizards, such as diverse vegetation (food resources), shelter (groups of large rocks), and suitable substrates for egg-laying. However, unsuccessful reintroductions may have been caused by incomplete predator eradication, disease acquired after release, or low reproduction rates insufficient to establish a stable population.

### 3.4. Predator Controls

For several years, feral cat control campaigns were implemented on each island. To that end, traps were placed in (or near) the original habitats and at sites where lizards had been reintroduced. Campaigns between 2001 and 2024 on El Hierro resulted in a total of 472 cats trapped: 139 in Risco Tibataje, 182 in La Dehesa, 143 in El Julan, 6 in Punta de Arelmo, 1 in Punta Agache, and 1 in Punta Miguel. These campaigns were halted in 2024 due to restrictions included in the Spanish Law on Animal Welfare.

On La Gomera, between 2002 and 2015, 220 cats were captured in the vicinity of Quiebracanillas (the base of the original habitat on the La Merica inland cliff), while 14 feral cats were also captured between 2018 and 2024 at the same site. One of the latter was captured three times, and, as it was identified, it was returned to the owners (with the recommendation that it should be kept indoors). Voluntary cat sterilisation campaigns were also publicised and carried out in local human communities of both islands. Gesplan, a public enterprise of the Autonomous Canarian Government, has been responsible for implementing the cat-trapping campaign in the last couple of years.

It is well known that cats are effective lizard predators [85,86], including endangered Canarian lizards [87]. For *G. bravoana*, some fences were placed near the original habitat and in one of the first reintroduction sites to keep cats out, but they were apparently not successful [17]. Actions have been taken in several parts of the world to mitigate the impact of cats on local fauna [88]; in Australia, these have included trapping, lethal baiting, and even shooting [89]. Lethal baiting has been greatly restricted in Australia, and the most frequently used protocol to control cat populations has been the Trap–Neuter–Return (TNR) strategy [89,90].

### 3.5. Results of Veterinary Controls

#### 3.5.1. General Sanitary Aspects

The main goal was to understand the species’ physiology to prevent, diagnose, and treat health issues, ensuring successful recovery actions [91]. Comparative haematology, including cytology and blood chemistry [92,93], was performed for both species, revealing strong physiological resilience to arid, low-rainfall environments. Plasma ions and renal markers remained above typical levels for lizards from humid climates, indicating specific adaptations and notable resistance to arid conditions [94].

Studies on cloacal microbiology in both lizard species showed a lower proportion of Gram-positive than Gram-negative bacteria [95]. Identified genera included *Citrobacter*, *Corynebacterium*, *Enterobacter*, *Enterococcus*, *Escherichia*, *Klebsiella*, *Pseudomonas*, *Salmonella*, *Staphylococcus*, and *Streptococcus*, while *Aspergillus*, *Candida*, and *Rhodotorula* were the only fungi and yeasts detected. These microorganisms are considered normal in healthy lizards, though some may be potentially pathogenic, highlighting the importance of host immune status in disease development. Table 7 summarises the health-related studies for each species.

The analyses independently performed on each species were as follows:

#### 3.5.2. *Gallotia simonyi*

Lipophilic analyses of femoral gland secretions identified 57 compounds, including steroids (mainly cholesterol), fatty acids (hexadecanoic and octadecanoic acids), aldehydes, alcohols, ketones, squalene, and wax esters. Males showed higher proportions of odoriferous compounds such as fatty acids and aldehydes, while females had more stable compounds like steroids, waxy alcohols, wax esters, and terpenoids. These results improved understanding of the species’ chemical communication and may clarify mechanisms of mate choice or hierarchy formation in the wild [96].

Disease analyses were carried out when needed. Some individuals developed oral tumours later identified as benign gingival hyperplasia [98]. Faecal samples were examined for bacteriological and coprological studies, leading to the detection of a new Salmonella serovar (*Salmonella bongori* 13,22:z39:–) in the El Hierro Giant Lizard [99]. Nematodes were also naturally present, but without disrupting the parasite–host balance in any of the animals analysed.

#### 3.5.3. *Gallotia bravoana*

The first individuals captured for the recovery programme exhibited maladaptation syndrome [92], and a high prevalence of erythrocyte protozoa was detected in them [100], all associated with adaptation processes to new conditions and the stress of captivity. Once these issues were corrected, cardiac physiology for this species was established based on electrocardiogram parameters in the well-adapted animals [97].

#### 3.5.4. Health Conclusions

The haematological and biochemical study characterised blood cell types in these species and showed that they tolerate unusually high levels of dehydration compared to continental lizards. Microbiological analyses revealed seasonal cycles in the main bacteria and fungi of the digestive tract and skin, with Gram-negative flora predominating and fluctuating throughout the year. Fungal populations also varied seasonally, peaking in spring, while increased summer sunlight reduced their growth and resulted in the lowest isolation rates.

During 2001–2004, some lizards—particularly those from La Gomera—showed low calcium:phosphorus ratios, low plasma ionised calcium levels, and poor skeletal mineralisation, attributed to secondary hyperparathyroidism of renal or nutritional origin. This condition prompted close monitoring due to its impact on the breeding programme. In recent years, preventive measures, including supplements and balanced nutrition, were implemented, and calcium-related problems did not recur.

To maintain equilibrium with internal parasites, prophylactic antiparasitic treatment with metronidazole and fenbendazole has proven effective, both during captivity and in individuals intended for release into the wild. Finally, mite outbreaks—mainly Ophionyssus galloticolus—occurring in wet years required updated management and disinfection protocols to avoid health impacts.

### 3.6. Educational Activities

For several years, different educational activities related to each species have been performed with children and adolescents of several schools on each island, although the general public has also been invited. In Table 8, we include the years and the type of audience receiving information on the history and conservation actions related to each species in these events.

Public visits to an interpretation centre in Frontera (El Hierro) were established in 1995. In Table 9, we include the available data since 2006 on the number of visitors per year. At present, there is no interpretation centre on La Gomera; however, the breeding centre has received student visits since 2006 (Table 9).

In general, participants in these activities showed strong interest in learning about these lizards and the history of each species, including their maintenance in terraria for breeding and the efforts to reintroduce them into natural habitats.

## 4. Conclusions

Females and males of both *G.simonyi* and *G. bravoana* have been maintained in breeding centres located on the islands of El Hierro and La Gomera, respectively, in the Canary Archipelago for several decades. They have been kept in outdoor terraria at each centre, and different mating pairs were established annually to obtain offspring that could eventually participate in reintroduction trials involving adult lizards. Breeding during the past 19 years has resulted in a current population of 216 individuals of *G. simonyi* and 330 of *G. bravoana*. The number of eggs laid by females and the number of successfully hatched offspring have varied over this period, but without evidence of a clear trend. Both female and male SVL showed a significant relationship with the number of eggs laid and the number of successfully hatched offspring. Only certain specific reproductive pairs influenced female fecundity and hatching success.

Several reintroductions of individuals from each species into different localities on their respective islands have generally not resulted in the establishment of viable populations. In El Hierro, aside from the species’ original habitat, only a stable population of *G. simonyi* (approximately 126 individuals) persists on a small islet near the north-western shore, and an unknown number of reintroduced lizards remain in an inland locality. In La Gomera, aside from the original habitat of *G. bravoana*, an unknown number of individuals persist following a recent inland reintroduction. The next census on each island will aim to estimate the number of surviving individuals in these inland sites. Nevertheless, it is noteworthy that both species remain endangered; therefore, increased investment in personnel and infrastructure at both breeding centres is urgently needed to improve management and ensure faster recovery of each species. Special attention must be given to maintaining cat-control campaigns, at least in or near original habitats and reintroduction sites.

Veterinary monitoring of individuals from both species showed that they tolerate unusually high levels of dehydration, while their microbial flora—mainly Gram-negative bacteria and seasonally fluctuating fungi—vary throughout the year. Health assessments revealed past calcium–phosphorus imbalances in some populations, which were resolved through improved nutrition and supplementation. Effective parasite control and updated management protocols, particularly during mite outbreaks in unusually wet years, have contributed to maintaining overall animal health.

## Figures and Tables

**Table 1:** GLM statistics parameters obtained for the analyses of the number of eggs laid considering species, year, and male and female SVL as covariates (**a**) and eliminating the male SVL covariate (**b**).

(**a**)			
**Origin**	**Wald’s Chi-2**	**df**	***p***
(Intersection)	0.864	1	0.353
Species	9.795	1	0.002
Year	37.592	16	0.002
Male SVL	0.703	1	0.402
Female SVL	12.530	1	0.014
AIC: 1762.44			
(**b**)			
**Origin**	**Wald’s Chi-2**	**df**	***p***
(Intersection)	1.732	1	0.188
Species	14.315	1	<0.001
Year	40.772	16	<0.001
Female SVL	12.530	1	<0.001
AIC: 1761.13			

**Table 2:** Parameters of GLM using HO as the dependent variable, with (**a**) or without (**b**) species as one of the factors.

(**a**)			
**Origin**	**Wald’s Chi-2**	**df**	***p***
(Intersection)	4.558	1	0.033
Species	0.705	1	0.401
Year	47.606	16	<0.001
Male SVL	7.031	1	0.008
Female SVL	6.342	1	0.012
AIC: 2050.10			
(**b**)			
**Origin**	**W** **ald’s Chi-2**	**df**	***p***
(Intersection)	22.092	1	<0.001
Year	48.095	16	<0.001
Male SVL	10.895	1	<0.001
Female SVL	9.409	1	0.002
AIC: 2048.80			

**Table 3:** GLM statistics parameters obtained for the analyses of the number of laid eggs (**a**) and number of hatched offspring (**b**) of the two species, considering male-to-female difference in SVL as the only covariate.

(**a**)			
**Origin**	**Wald’s Chi-2**	**df**	***p***
(Intersection)	1483.331	1	<0.001
Species	106.005	1	<0.0001
Year	48.914	16	<0.0001
Male-to-female SVL			
difference	0.024	1	0.877
(**b**)			
**Origin**	**Wald’s Chi-2**	**df**	***p***
(Intersection)	707.794	1	<0.001
Species	72.464	1	<0.001
Year	46.801	16	<0.001
Male-to-female SVL			
difference	1.510	1	0.219

**Table 4:** Cook distances for specific male–female reproductive pairs with greater influence on the dependent variables in *G. simonyi* (**a**) and *G. bravoana* (**b**). Codes in bold signal those pairs having greater influence on one or two dependent variables.

(**a**)			
**Male Code**	**Female Code**	**Cook’s Distance**	**Cook’s Distance**
		(NEL)	(HO)
**0051**	**0A48**		0.099
**3471**	**5935**	0.027	0.062
5330	1518		0.053
0333 f	633A		0.050
0235 (cf 0230)	0917		0.049
9578	4858		0.042
1430	6919	0.041	
**0050**	**4858**		0.041
9457	7594		0.041
4330	6919	0.040	
5351	0010(433A)		0.039
**0051**	**0A48**	0.038	
9850	2821		0.038
086B	hembra 1	0.037	
0140F	0A48	0.036	
3-4554	4858	0.030	
0050	0100	0.023	
4040	7C70	0.017	
**0050**	**4858**	0.016	
(**b**)			
**Male Code**	**Female Code**	**Cook’s Distance**	**Cook’s Distance**
		(NEL)	(HO)
1624	4376		0.169
**8092**	**374D**		0.154
8195	5945	0.139	
7903	5076		0.116
**8250**	**0886**		0.108
**8092**	**374D**	0.098	
8084	3405		0.080
0886	374D	0.060	
5677	5743	0.057	
1467	1185	0.057	
6049	5280	0.052	
9998	2258		0.052
8250	1185	0.051	
**8250**	**0886**	0.050	
O517	3349	0.048	
8299	0313	0.047	
8195	8810		0.046
**1467**	**1185**		0.045
2218	5511		0.044
1503	6025		0.042

**Table 5:** Results from GLM analysis testing the effect of year on NEL (**a**) and on HO (**b**) of *G. simonyi*. Years in bold correspond to those having significantly higher or lower NEL or HO.

(**a**)				
	**Exp (B)**	**Wald’s Chi-2**	**df**	***p***
(Intersection)	10.733	906.882	1	<0.001
**2006**	0.642	8.802	1	0.003
**2007**	0.755	4.27	1	0.039
2008	0.820	2.244	1	0.134
**2009**	0.776	3.951	1	0.047
2010	0.815	2.653	1	0.103
**2011**	0.765	4.799	1	0.028
**2012**	0.571	11.822	1	<0.001
**2013**	0.673	7.268	1	0.007
**2014**	0.719	7.051	1	0.008
**2015**	0.677	9.889	1	0.002
2016	0.857	1.766	1	0.184
2018	0.943	0.275	1	0.60
2019	0.826	2.659	1	0.103
2020	0.879	1.29	1	0.256
2022	0.990	0.009	1	0.927
2024	1			
(**b**)				
	**Exp (B)**	**Wald’s Chi-2**	**df**	***p***
(Intersection)	6.533	345.236	1	<0.001
2006	0.782	1.887	1	0.17
2007	0.949	0.104	1	0.747
2008	1.163	0.979	1	0.322
2009	0.969	0.041	1	0.839
2010	1.250	2.440	1	0.118
2011	1.006	0.002	1	0.968
**2012**	0.670	4.147	1	0.042
2013	0.850	0.87	1	0.351
2014	1.050	0.113	1	0.736
**2015**	0.571	11.160	1	<0.001
2016	1.071	0.241	1	0.623
2018	1.014	0.01	1	0.921
2019	0.990	0.005	1	0.943
2020	1.071	0.249	1	0.618
2022	0.985	0.011	1	0.917
2024				

**Table 6:** Results from GLM analysis testing the effect of year on NEL (**a**) and on HO (**b**) of *G. bravoana.* Years in bold correspond to those having significantly higher or lower NEL or HO.

(**a**)				
	**Exp (B)**	**Wald’s Chi-2**	**df**	***p***
(Intersection)	4.615	140.343	1	<0.001
**2009**	0.617	5.349	1	**0.021**
2010	0.831	0.898	1	0.343
2011	0.985	0.01	1	0.92
2012	1.333	2.838	1	0.092
**2018**	1.458	4.704	1	0.03
**2019**	1.50	5.918	1	**0.015**
2022	1.288	2.462	1	0.117
2023	1.346	3.142	1	0.076
2024	1			
(**b**)				
	**Exp (B)**	**Wald’s Chi-2**	**df**	***p***
(Intersection)	2.308	20.979	1	<0.001
**2009**	0.267	11.034	1	<0.001
2010	1.336	1.391	1	0.238
2011	1.392	2.557	1	0.11
**2012**	2.333	15.076	1	<0.001
**2018**	2.403	15.458	1	<0.001
2019	2.133	11.726	1	<0.001
2022	1.878	8.602	1	0.003
2023	1.671	5.089	1	0.024

**Table 7:** Tests performed in different years and on randomly selected individuals of Gs (*Gallotia simonyi*) and Gb (*Gallotia bravoana*).

Test/Study	Year	Species	Results
		(Gs, Gb)	
Microbiology	2003	Gs + Gb	Published data
			New Salmonella (Martínez-Silvestre et al., 2003; Herrera-León et al., 2005)
PCR (Arenavirus, Reovirus, Ranavirus)	2022, 2024, 2025	Gs + Gb	Negative [non-published]
Haematology	2001, 2002, 2003, 2004	Gs + Gb	Published data [92,93,94]
Femoral gland			
cromatography		Gs	Published data [96]
Electrocardiography	2003	Gb	Published data [97]
	2022, 2024, 2025	Gs	
Necropsies and	2004, 2022,	Gs + Gb	Published data [98]
histology	2025		

**Table 8:** Divulgation talks given on the endangered giant lizards of La Gomera and El Hierro between 2019 and 2024.

Year	Centre	Audience
2019	Cabildo El Hierro	Environmental agents
2019	Cabildo El Hierro	Touristic guides (Interpretation Centre)
2019	C.E.I.P. Ruiz de Padrón	
	(La Gomera)	40 children (primary 3rd and 4th)
2024	C.E.O. Nereida Díaz Abreu	
	(La Gomera)	120 children (primary. 1st, 2nd, 3rd)
2024	I.E.S. San Sebastián (La Gomera)	60 children (1st year Obligatory Secondary Teaching)
2024	C.E.I.P. Tigaday (El Hierro)	90 children (primary 3rd and 4th)
2024	Casa de la Cultura La Gomera	General public
2024	Centre Initiatives Tourism, El Hierro	General public

**Table 9:** Number of people per year visiting Frontera Interpretation Centre since 2006. Source: Empresa Insular de Servicios Meridiano.

					*G. simonyi*
Number of People	Year	Number of People	Year	Number of People	Year
10,246	2020	10,375	2013	13,128	2006
12,754	2021	10,655	2014	11,552	2007
16,744	2022	12,999	2015	10,576	2008
18,005	2023	12,350	2016	7934	2009
21,014	2024	15,122	2017	7477	2010
20,753	2025	21,528	2018	7773	2011
		17,134	2019	8520	2012
					*G. bravoana*
0	2020	79	2013	130	2006
30	2021	130	2014	185	2007
67	2022	80	2015	230	2008
72	2023	135	2016	350	2009
67	2024	75	2017	640	2010
82	2025	66	2018	380	2011
		85	2019	440	2012

## Data Availability

Restrictions apply to the availability of these data. Data were obtained directly from authors and collaborators. Data reported in the current review are not deposited in any repository. However, specific data may be available from the authors on request.

## Supplementary Materials

The following supporting information can be downloaded at https://www.mdpi.com/article/10.3390/ani16121869/s1. Table S1: Basic statistics for female and male SVL of *G. simonyi* (a) and *G. bravoana* (b) of every year; Table S2: Mean (±S.E.), minimum, maximum values, sample size (N) and total number (T) for NEL and HO in each of the years analysed for *G. simonyi* and *G. bravoana*; Table S3: Actions and number of reintroduced lizards (total) in different natural habitats and along years for *G. simonyi* (a) and *G. bravoana* (b). For some sites and years there are no number of individuals but some observational data.
