# Supplementary material for: Long-Term Conservation Management of the Endangered Canarian Lizards Gallotia simonyi and G. bravoana (Fam. Lacertidae) (2006–2024)
Source: Animals (Basel). 2026 Jun 17;16(12):1869. doi: 10.3390/ani16121869 (PMC13295717; doi:10.3390/ani16121869)
Supplement: Supplementary file 1 [file animals-16-01869-s001.zip › animals-4210800-supplementary.pdf]

Supplementary material

Table S1. Basic statistics for female and male SVL of *G. simonyi* (a) and *G. bravoana* (b) of every year.

a)

Species	Year	Statistics	Female Snout-vent length (mm)	Male Snout-vent length (mm)
G. simonyi	2006	Mean	190.89	207.22
		S.E.	3.518	4.561
		Minimum	176	186
		Maximum	205	226
		N	9	9
	2007	Mean	195.70	215.30
		S.E.	4.88	6.07
		Minimum	159	182
		Maximum	212	246
		N	10	10
	2008	Mean	194.40	215.50
		S.E.	4.78	5.99
		Minimum	159	182
		Maximum	212	246
		N	10	10
	2009	Mean	200.92	211.17
		S.E.	4.28	5.77
		Minimum	164	182
		Maximum	219	246
		N	12	12
	2010	Mean	194.92	218.17
		S.E.	3.14	4.49
		Minimum	176	192
		Maximum	210	247
		N	12	12
	2011	Mean	193.64	223.43
		S.E.	2.93	6.10
		Minimum	174	200
		Maximum	210	288
		N	14	14
	2012	Mean	193.50	222.13
		S.E.	4.47	4.22
		Minimum	175	202
		Maximum	211	235
		N	8	8
	2013	Mean	195.13	226.50
		S.E.	3.74	3.62
		Minimum	185	215
		Maximum	214	241
		N	8	8
	2014	Mean	192.14	223.93
		S.E.	2.64	2.72
		Minimum	175	210
		Maximum	207	240
		N	14	14
	2015	Mean	194.53	223.60
		S.E.	2.43	2.54
		Minimum	181	211
		Maximum	216	242
		N	15	15
	2016	Mean	192.0	225.42

		S.E.	2.46	2.89
		Minimum	175	211
		Maximum	207	240
		N	15	12
	2018	Mean	200.20	225.73
		S.E.	2.29	3.07
		Minimum	187	201
		Maximum	218	245
		N	15	15
	2019	Mean	198.47	225.73
		S.E.	2.25	3.07
		Minimum	187	201
		Maximum	218	245
		N	15	15
	2020	Mean	201.60	226.75
		S.E.	3.02	3.67
		Minimum	187	201
		Maximum	218	243
		N	10	12
	2022	Mean	198.88	227.00
		S.E.	2.23	2.57
		Minimum	187	201
		Maximum	218	243
		N	16	16
	2024	Mean	201.20	227.87
		S.E.	2.20	2.49
		Minimum	186	211
		Maximum	218	243
		N	15	15
		Total N	198	198

b)

G. bravoana	2009	Mean	162.15	175.15
		S.E.	2.67	2.34
		Minimum	151	165
		Maximum	185	198
		N	13	13
	2010	Mean	161.17	172.75
		S.E.	3.09	2.80
		Minimum	143	163
		Maximum	186	198
		N	12	12
	2011	Mean	160.48	175.70
		S.E.	1.46	1.35
		Minimum	149	167
		Maximum	185	200
		N	33	33
	2012	Mean	163.46	181.08
		S.E.	1.52	1.24
		Minimum	155	176
		Maximum	178	193
		N	13	13
	2018	Mean	164.73	182.09
		S.E.	1.67	2.23
		Minimum	158	175
		Maximum	177	198
		N	11	11
	2019	Mean	173.69	192.85
		S.E.	1.46	1.01
		Minimum	170	188
		Maximum	190	198
		N	13	13

2022	Mean	171.27	189.69
	S.E.	1.09	0.97
	Minimum	164	186
	Maximum	180	199
	N	15	13
2023	Mean	156.73	173.00
	S.E.	1.30	1.79
	Minimum	146	159
	Maximum	162	184
	N	11	11
2024	Mean	171.58	190.25
	S.E.	1.39	1.45
	Minimum	164	181
	Maximum	180	199
	N	12	12
	Total N	133	133

Table S2. Mean (± S.E.), minimum, maximum values, sample size (N) and total number (T) for NEL and HO in each of the years analysed for *G. simonyi* and *G. bravoana*.

Species	Year	Variable	Mean	S.E.	Minimum	Maximum	N	T
G. simonyi	2006	NEL	6.89	0.857	1	10	9	62
		HO	5.11	1.274	0	10	9	46
	2007	NEL	8.1	0.379	7	10	10	81
		HO	6.2	1.073	0	9	10	62
	2008	NEL	8.8	0.554	6	12	10	88
		HO	7.6	1.118	1	12	10	76
	2009	NEL	9	0.775	7	12	12	--
		HO	6.33	1.257	0	11	12	76
	2010	NEL	8.75	0.463	5	12	12	105
		HO	8.17	0.458	5	10	12	98
	2011	NEL	9	0.365	8	10	6	54
		HO	8.5	0.5	7	10	6	51
	2012	NEL	6.13	0.915	2	11	8	49
		HO	4.37	1.362	0	11	8	35
	2013	NEL	7.22	0.434	6	9	9	65
		HO	5.56	0.899	0	8	9	50
	2014	NEL	7.71	0.507	4	11	14	108
		HO	6.86	0.702	1	10	14	96
	2015	NEL	7.27	0.881	0	12	15	109
		HO	3.73	1.119	0	11	15	56
	2016	NEL	9.2	0.841	0	14	15	138
		HO	7	0.995	0	12	15	105
	2018	NEL	10.13	0.769	5	15	16	162
		HO	6.62	1.245	0	15	16	106

	2019	NEL	8.87	0.92	0	14	15	133
		HO	6.47	1.133	0	11	15	97
	2020	NEL	9.44	0.724	2	14	16	151
		HO	7	1.057	0	13	16	112
	2022	NEL	10.63	0.87	1	16	16	170
		HO	6.44	1.255	0	14	16	103
	2024	NEL	10.73	0.968	7	19	15	161
		HO	6.53	1.187	0	15	15	98
	Total	NEL	8.8	0.215	0	19	192	1690
		HO	6.4	0.284	0	15	192	1267
G. bravoana	2009	NEL	2.85	0.758	0	9	13	37
		HO	0.62	0.331	0	4	13	8
	2010	NEL	3.83	0.842	1	10	12	46
		HO	3.08	0.866	0	10	12	37
	2011	NEL	4.55	0.528	0	9	33	106
		HO	3.21	0.503	0	9	33	33
	2012	NEL	6.15	0.504	2	8	13	80
		HO	5.38	0.626	0	8	13	70
	2018	NEL	6.73	1.001	1	11	11	74
		HO	5.55	1.056	0	10	11	61
	2019	NEL	6.92	0.895	1	11	13	90
		HO	4.92	0.604	1	9	13	64
	2022	NEL	5.94	0.475	3	10	18	107
		HO	3.89	0.512	0	6	18	35
	2023	NEL	6.21	0.459	3	9	14	87
		HO	3.82	0.989	0	8	14	42
	2024	NEL	4.58	1.003	0	10	12	55
		HO	1.63	1.068	0	7	12	13
	Total	NEL	5.22	0.251	0	11	139	796
		HO	3.54	0.268	0	10	139	499

Table S3. Actions and number of reintroduced lizards (total) in different natural habitats and along years for *G. simonyi* (a) and *G. bravoana* (b). For some sites and years there are no number of individuals but some observational data.

a)

Species	Site	Year	Total	Estimated number of lizards	Reference
G. simonyi					
	Roque Chico Salmor	1999	21		Rodríguez-Domínguez et al. [61]
		2001	15		Rodríguez-Domínguez et al. [61]
		2003		number of lizards: 36 (30-45)	Silva et al. [62]
		2005		Schnabel estimation: 40 lizards	González-Ortega et al. [64]
		2005	5-40		Rodríguez-Domínguez et al. [61]
		2008		Population size evaluation: 126 lizards	Trujillo [65]
	La Dehesa	2001	88		Silva et al. [62]
		2003		Estimation of 36 lizards	Silva et al. [62]
		2004		few or no detection of living lizards	Molina-Borja et al. [63]
		2005	22		Rodríguez-Domínguez et al. [61]
		2005		33-37 lizards (Schnabel method)	González-Ortega et al. [64]
		2008		Schnabel estimation: 1.3 (0.5-3.0)	Trujillo [65]
	El Julan	1999	12		Silva et al. [62]
		2000	153		Silva et al. [62]
		2001	63		Silva et al. [62]
		2003		estimation of 15 lizards (heavy rains:	Silva et al. [62]
		2004		few or no detection of lizards	Molina-Borja et al. [63]
		2005	20		Rodríguez-Domínguez et al. [61]
		2005		estimation of 28-29 lizards	González-Ortega et al. [64]
		2008		4 lizards captured; no recapture= no estimation	Trujillo [65]

Risco Tibajate: (original habitat)	1997		Estimated maximum population size: 1613 lizards in 56953 m^2 Jolly-Seber method)	Rodríguez-Domínguez et al. [61]
	1999		327 lizards: estimated maximum population size in 36605 m^2	Pérez-Mellado et al. [9]
	2007		Population size evaluation: 345 lizards	Trujillo [58]
R. Tibataje	2019		Population size evaluation: 834 individuals	Martín-Carbajal and Martínez-Iglesias [59]
C. Agache	2013	32		Staff Environmental Council, Cabildo El Hierro
C. Agache	2019		75 recent lizard excrements found	Martín-Carbajal and Padilla, [66]
C. Agache	2020	50		Martín-Carbajal [67]
C. Agache	2024	71	evaluation in that population will be performed along 2026	Martín-Carbajal and Padilla, [68]
P. Arelmo	2013	35		Staff of Traverca Enterprise: service paid for by Cabildo El Hierro
P. Arelmo	2014		some fresh excrements found	Pérez-Padilla, pers. commun.
P. Miguel	2016	46		Portero-Alvarez and Martín-Carbajal [69]
	2018		April-june 2018: no lizard, no activity, no excrements	
	Total:	629		

b)

G. bravoana

Original habitat:			
La Mérica cliff	2004	Estimated number of lizards: 80	Mateo [11]
Los Órganos	2008	Experimental reintroduction of 6 individuals	Mateo [11]
Original habitat:			
La Mérica cliff	2009	Around 160 lizards in an area of ca 2 km2	Curbero [74]

Original habitat:				
La Mérica cliff	2016		Reduction in recent lizard excrements found in sites of the original population	Martín-Carbajal and Portero-Alvarez [75]
Original habitat:				
La Mérica cliff	2022		Sampling different sites of La Merica cliff estimated number of lizards: 92	Padilla and Martín-Carbajal [76]
Los Órganos	2014	135		Romero; staff Cabildo La Gomera
Los Órganos	2015		No lizards in traps, no fresh excrements	Romero; staff Cabildo La Gomera
Los Órganos	2016		2016: no lizard or signs of activity detected	Martín-Carbajal and Portero-Alvarez [75]
Oroja	2013	18		Romero; staff Cabildo La Gomera
Oroja	2016	39		Martín-Carbajal and Portero-Alvarez [75]
	2024		No sign of lizard presence	Martín-Carbajal and Padilla (pers. commun.)
Pta. Salinas	2016	105		Martín-Carbajal and Portero-Alvarez [75]
Pta. Salinas	2018		no lizard, no activity, no excrements neither in Oroja nor in Pta. Salinas	Martín-Carbajal (pers. Comm.)
Pta. Salinas	2024		No sign of lizard presence	Martín-Carbajal and Padilla (pers. comm.)
Quiebracanillas	2016		Fresh lizard excrements found	Martín-Carbajal and Portero-Alvarez [75]
Quiebracanillas	2022		no presence of lizards in this area	Bohórquez-Alonso and Molina-Borja [90]
C. Heredia	2022	83		Padilla and Martín-Carbajal (in prep.)
	Total:	380		

After 2022 new data have been gathered in this last site, and they are currently being processed
